# Progression of pelvic retroversion is a critical factor for clinical outcome after Opening-wedge high tibial osteotomy among elderly patients

**DOI:** 10.1186/s40634-021-00376-5

**Published:** 2021-08-18

**Authors:** Shuhei Otsuki, Hitoshi Wakama, Kuniaki Ikeda, Nobuhiro Okuno, Yoshinori Okamoto, Tomohiro Okayoshi, Junya Matsuyama, Masashi Neo

**Affiliations:** Department of Orthopedic Surgery, Osaka Medical and Pharmaceutical University, 2-7 Daigakumachi Takatsuki, Osaka, 569-8686 Japan

## Abstract

**Purpose:**

To evaluate the clinical outcome after opening-wedge high tibial osteotomy (OWHTO) and to determine the critical factors for a poor clinical outcome after OWHTO in patients aged over 65 years.

**Methods:**

Our retrospective analysis was based on the data from 233 patients who underwent OWHTO for medial compartment knee OA at our institution between January 2013 and December 2018, and 88 patients (36 men and 52 women) over 65 years of age were included in this study. Radiographic parameters of weight-bearing line ratio (WBLR) and pelvic inclination (PI); the knee function, range of motion (ROM) and extension; and clinical outcome with Lysholm score were obtained preoperatively and postoperatively at the final follow-up visit. To evaluate the critical factors for the clinical outcome, univariate regression analysis was used to identify the relationship between postoperative and improved Lysholm score and pre-and post-operative essential factors. To reveal the factor having a greater impact on the clinical outcome, a *p* < 0.05 in univariate factors was entered into a multivariate regression analysis.

**Results:**

The preoperative WBLR was significantly changed, and Lysholm score improved from 59.5 to 81.5 (*p* < 0.0001), whereas the PI, knee extension and ROM were not changed after OWHTO. Regarding the essential factors affecting clinical outcome after OWHTO, age and delta PI were negative, whereas preoperative WBLR, postoperative ROM, especially extension, had a positive effect (*p* < 0.05). Furthermore, only delta PI had affected the improvement of clinical outcome with OWHTO (*p* < 0.01), and postoperative knee extension was negatively correlated with the progression of pelvic retroversion (*p* < 0.01).

**Conclusion:**

Age at surgery and progression of pelvic retroversion were the critical factors for poor postoperative clinical outcomes after OWHTO. Care should be taken for the progression of pelvic retroversion after OWHTO because it deteriorates the clinical outcome by inducing the knee flexion contracture as the compensatory mechanism for the balance of sagittal alignment.

## Background

Opening-wedge high tibial osteotomy (OWHTO) is a successful treatment for medial compartment knee osteoarthritis (OA) [1, 2, 19]. With recent advances in surgical technique, fixation device, and patient selection, OWHTO has yielded good clinical outcomes, especially for young and active patients with symptomatic medial knee compartment OA, and with a 10-year satisfaction rate of > 90% among patients < 50 years of age [5, 7]. Age is a risk factor for OWHTO and connotes a higher failure rate for patients over 65 years [18, 24]; however, some studies did not identify the effect of age on OWHTO [3, 4, 9]. Therefore, the effect of aging on the clinical outcome after OWHTO and its related factors remain an issue of controversy. In addition, the clinical outcome was generally evaluated postoperatively, which affected patients’ activity levels. The activity level of elderly patients is generally lower than that of younger patients, and the degree of improvement, measured from pre- to postoperative outcome, should be considered individually to exclude their own potential activity.

Spinal deformity is known to progress with aging, and changes in the sagittal plane alignment tend to advance spinal kyphosis and pelvic retroversion [21]. Sagittal malalignment is common among aging individuals, with restrictions in knee extension being apparent after the age of 60 [14]. According to Lafage et al., spinal kyphosis induces pelvic retroversion, hip extension, and knee flexion as compensatory mechanisms [11]. Poor global alignment negatively impacts the alignment of the lower extremities. Sagittal malalignment with pelvic retroversion affected knee OA via the effect on knee flexion contracture [10], which has been recognized to induce cartilage degeneration [16]. However, the effect of pelvic retroversion on the outcomes of OWHTO is still unclear. We hypothesized that the clinical outcomes after OWHTO in elderly patients would decline over time and that the progression of pelvic retroversion with flexion knee contracture would be a critical factor of poor postoperative outcome after OWHTO. Therefore, the purpose of our study was to evaluate the clinical outcome after OWHTO in patients aged over 65 years and to determine the critical factors for a poor clinical outcome after OWHTO.

## Materials and methods

### Statement of ethics

This study was approved by our Institutional Review Board, and written informed consent was obtained from all patients for surgery and for the use of their data for research and publication.

### Patient group

**Fig. 1:**
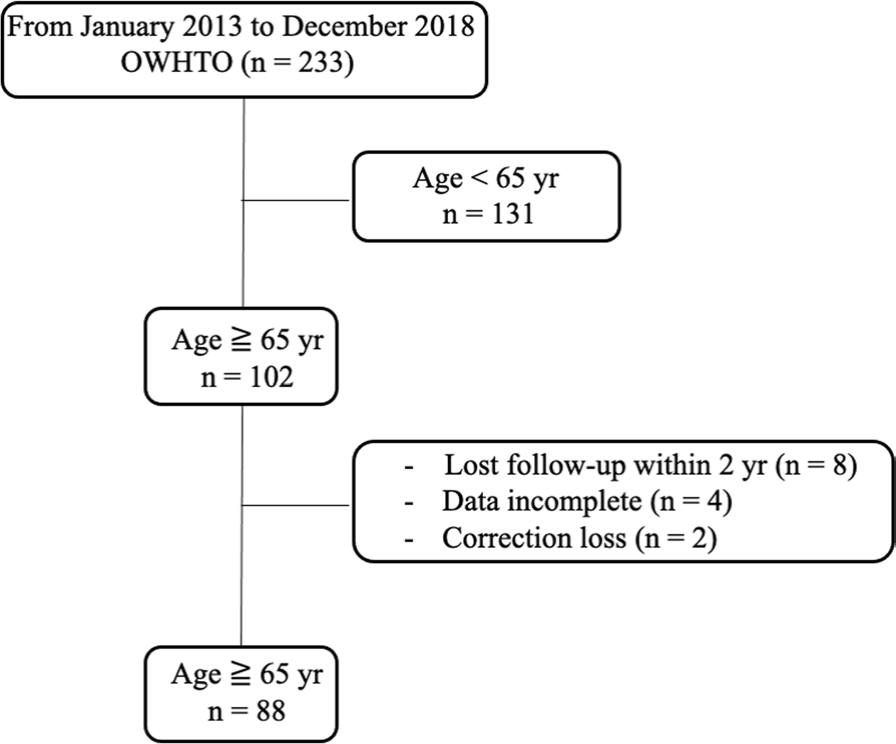
Flow chart of patient enrolment

Eligible were the 233 patients who underwent OWHTO for medial compartment knee OA at our institution between January 2013 and December 2018. The inclusion criteria for OWHTO among these patients were as follows: medial compartment knee OA or spontaneous osteonecrosis of the knee; requirement for a bony correction of < 15°, based on the preoperative planning; a body mass index (BMI) < 35 kg/m^2^; a preoperative knee flexion contracture ≤ 10º; and a minimum of 120º of knee ROM. The absence of, or well-controlled, diabetes mellitus was another inclusion criterion. Contraindications were as follows: symptomatic lateral compartment knee OA; symptomatic patellofemoral OA; a flexion contracture > 10°; and anterior or posterior cruciate ligament deficiency. A total of 131 patients aged > 65 years were excluded. Among these 102 patients, eight who were lost to follow-up within 2 years after OWHTO, four who did not undergo radiographic evaluation, and two who experienced a correction loss > 3° were excluded with hip-knee ankle angle. Therefore, 88 patients (32 men and 56 women) were included in our analysis (Fig. 1).

#### Surgical procedure and assessment

The aim of OWHTO is to correct the mechanical axis of the lower limb [12]. Preoperative planning for high tibial osteotomy (HTO) first considers the intended postoperative mechanical axis, which passes through the lateral tibial eminence on a coronal radiographic view. This axis was determined using digital planning software (TraumaCaD, BRAINLAB, Feldkirchen, Germany) in the Picture Archiving and Communication System (PACS). The surgical procedure used for OWHTO has been described elsewhere [17]. Briefly, the medial proximal tibia was exposed using a J-shaped incision, and the superficial medial collateral ligament and the pes anserinus were released. Biplane osteotomy is performed. Ascending cut was done 15 mm below the tibial tuberosity, and descending cut was referring to two Kirschner wires (K-wires) inserted into the proximal tibiofibular joint, 35 to 40 mm inferior to the knee joint line as the osteotomy guide. The gap created by the osteotomy was filled with β-tricalcium phosphate (OLYMPUS, Tokyo, Japan) and fixed using a locking plate (Tris Medial HTO Plate System; OLYMPUS, Tokyo, Japan).

#### Diagnostic and radiographic measurements

**Fig. 2:**
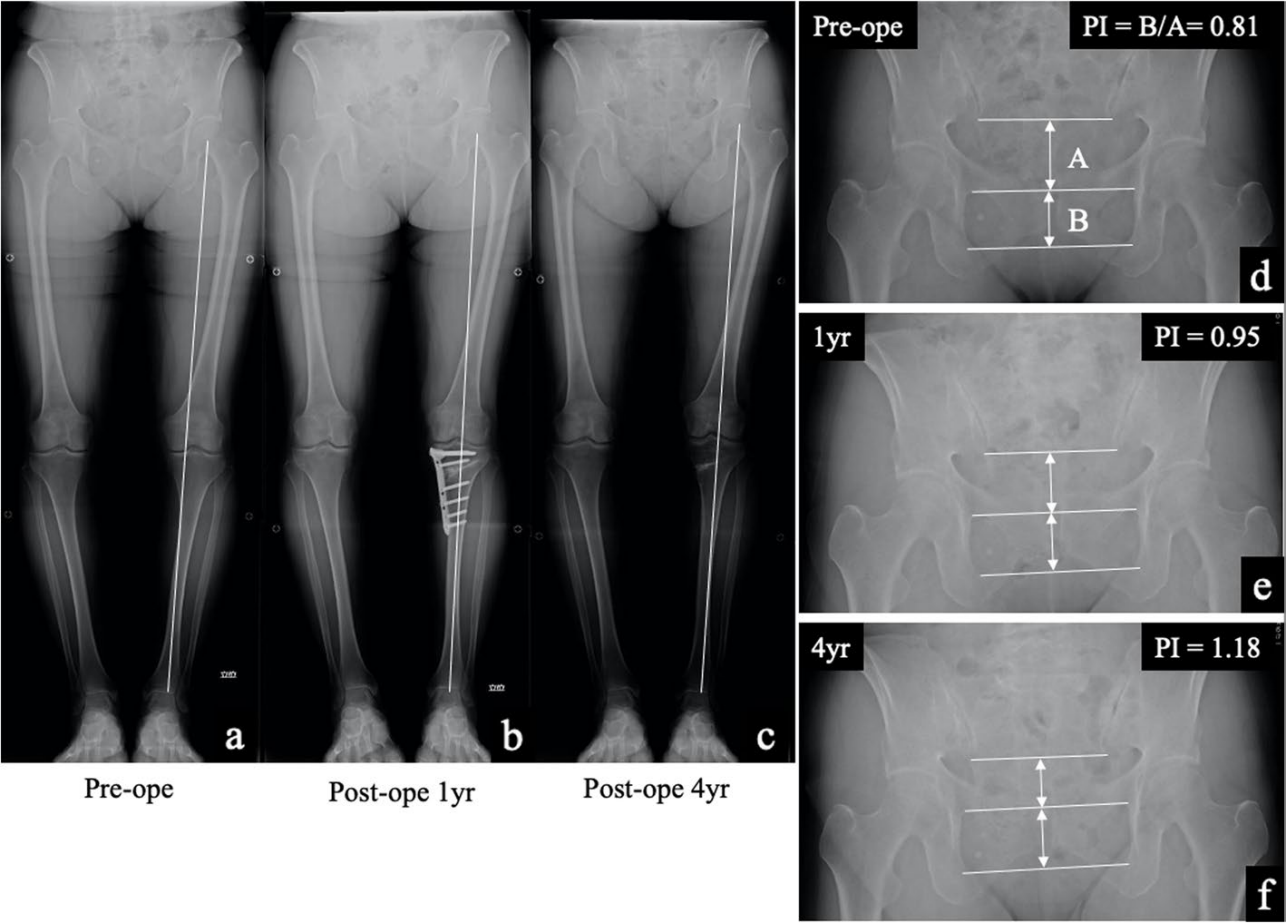
The pelvic inclination ratio. **a-c** Representative changes in pelvic inclination are shown. Representative whole-leg radiographs, in standing, are shown for a 78-year-old woman at the following time points: preoperatively and at 1 and 4 years after OWHTO, and at the final follow-up assessment. **d-f** Pre- to postoperative changes in the PI ratio are shown. The preoperative PI ratio of 0.81, 0.95 at 1 year after OWHTO and increased to 1.18 at the final follow-up

Pelvic inclination (PI) was assessed using the methods described by Schwartz et al. [23]. Pelvic radiographs in the neutral standing position were used, with the following two intermediate parameters drawn. The first line is drawn between the two lower margins of the sacroiliac joints, the second line is drawn between the upper borders of the two obturator foramens, and the third line is drawn between the two lower borders of the obturator foramens. The upper distance (A) is measured at the mid-point between the first and second line and correlates with the height of the lesser pelvis. The lower distance (B) is the height of the obturator foramen and is also measured at the mid-point between the second and the third line. The PI ratio was calculated as the B/A ratio. The representative changes in the PI ratio were shown in Fig. 2. The pre-and postoperative knee alignment was compared using the weight-bearing line ratio (WBLR). Specifically, the WBLR was measured as a line drawn from the center of the femoral head to the center of the ankle joint and the location where this line intersects with the tibial plateau, expressed as a percentage of the tibial width. All measurements were performed independently by two orthopedic surgeons, obtained prior to surgery and at the final follow-up. The knee function and clinical outcome were evaluated through the range of knee motion (ROM and extension) and Lysholm score, respectively.

#### Statistical analysis

A power analysis confirmed that 23 cases in each group were required, based on the evaluation of pre-and postoperative radiographic alignment and knee function using a two-sided Student’s *t*-test for statistical analysis, to yield a power of 0.8, a significance level of 0.05, and an effect size of 0.8 using G*power (version 3.1.9.2). Pre-, post- and improvement of Lysholm score between sex was analyzed by Mann–Whitney U-test. Univariate regression analysis was used to identify the relationship between postoperative and improvement of Lysholm score and pre-and post-operative predisposing factors. To reveal the factor having a greater impact on the clinical outcome, a *p* < 0.05 in univariate factors was entered into a multivariate regression analysis using the least-square method. The multicollinearity in multivariate analysis was obtained by calculating the variance inflation factor (VIF). Multicollinearity was set at VIF≧10. These analyses were performed using JMP Pro (version 15; SAS Institute Inc., Cary, NC), with a *p*-value < 0.05 considered significant. Inter- and intra-observer reliability for PI were evaluated using SPSS (Version 21.0; SPSS, Chicago, IL), with a *p-*value < 0.05 considered significant. The intra-observer and inter-observer reliability of the PI measurement were high at 0.994 (95% confidence interval [CI], 0.981–0.998) and 0.922 (95% CI, 0.765–0.975), respectively.

## Results

The average age was 72.4 ± 4.9 years, the mean BMI was 24.4 ± 2.8 kg/m^2^, and the average length of follow-up was 42.9 ± 13.5 (range, 26–84 months) after surgery (Table 1). Although the preoperative WBLR was significantly improved from 18.0 ± 13.1 to 57.9 ± 10.0% (*p* < 0.0001, Table 2), the PI and ROM were not changed after OWHTO. On the other hand, the Lysholm score was improved with OWHTO from 59.5 ± 9.0 to 81.5 ± 11.2 (*p* < 0.0001, Table 2). The essential factors affecting the clinical outcome after OWHTO were elucidated, and age and delta PI were negative, whereas preoperative WBLR, postoperative ROM and extension had a positive effect on the clinical outcome (*p* < 0.05, Table 3). Furthermore, the improvement of Lysholm score was compared with essential factors as well, and multivariate regression analysis showed that only delta PI had affected the improvement of clinical outcome with OWHTO without encountering multicollinearity (*p* < 0.05, Table 4). In addition, the correlation between delta PI and knee motion was elucidated, and postoperative knee extension was negatively correlated with delta PI (*p* < 0.05, Table 5).

**Table 1:**
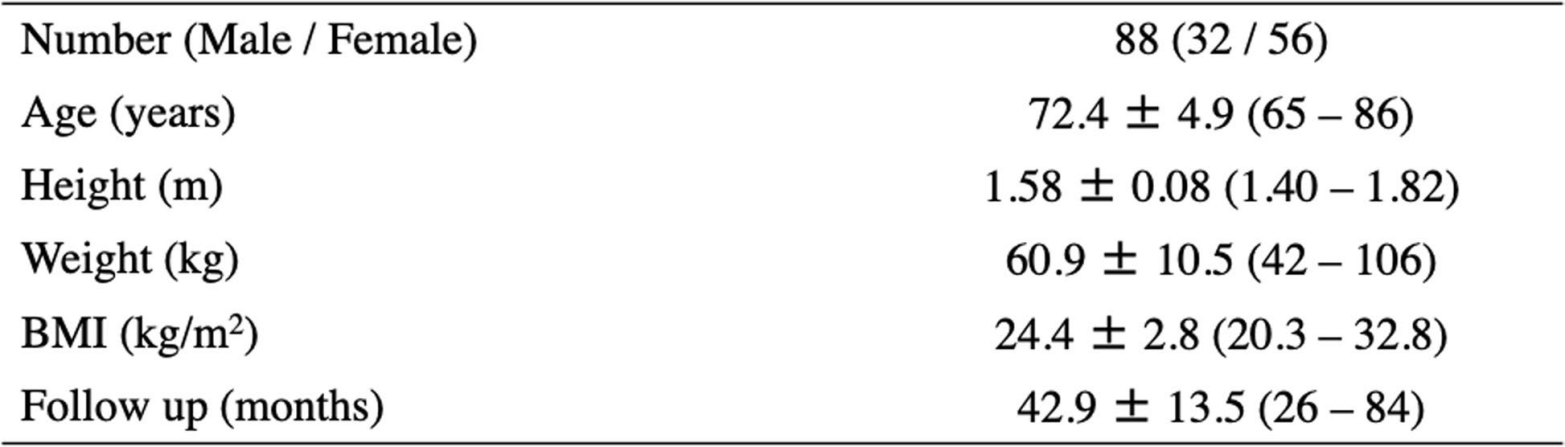
Demographic data

*BMI* body mass index, Data were shown mean ± standard deviation and range

**Table 2:**
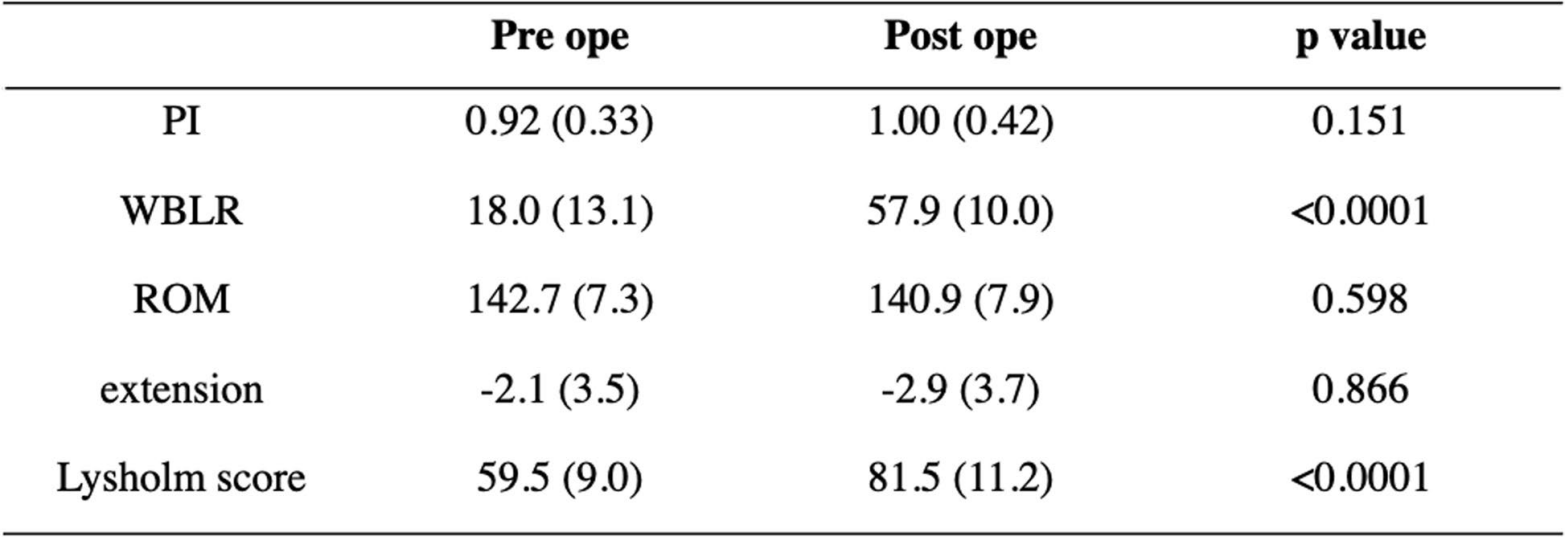
Changes of radiographic alignment and knee function

*PI* pelvic inclination, *WBLR* weight bearing line ratio, *ROM* range of motion (flexion – extension)

**Table 3:**
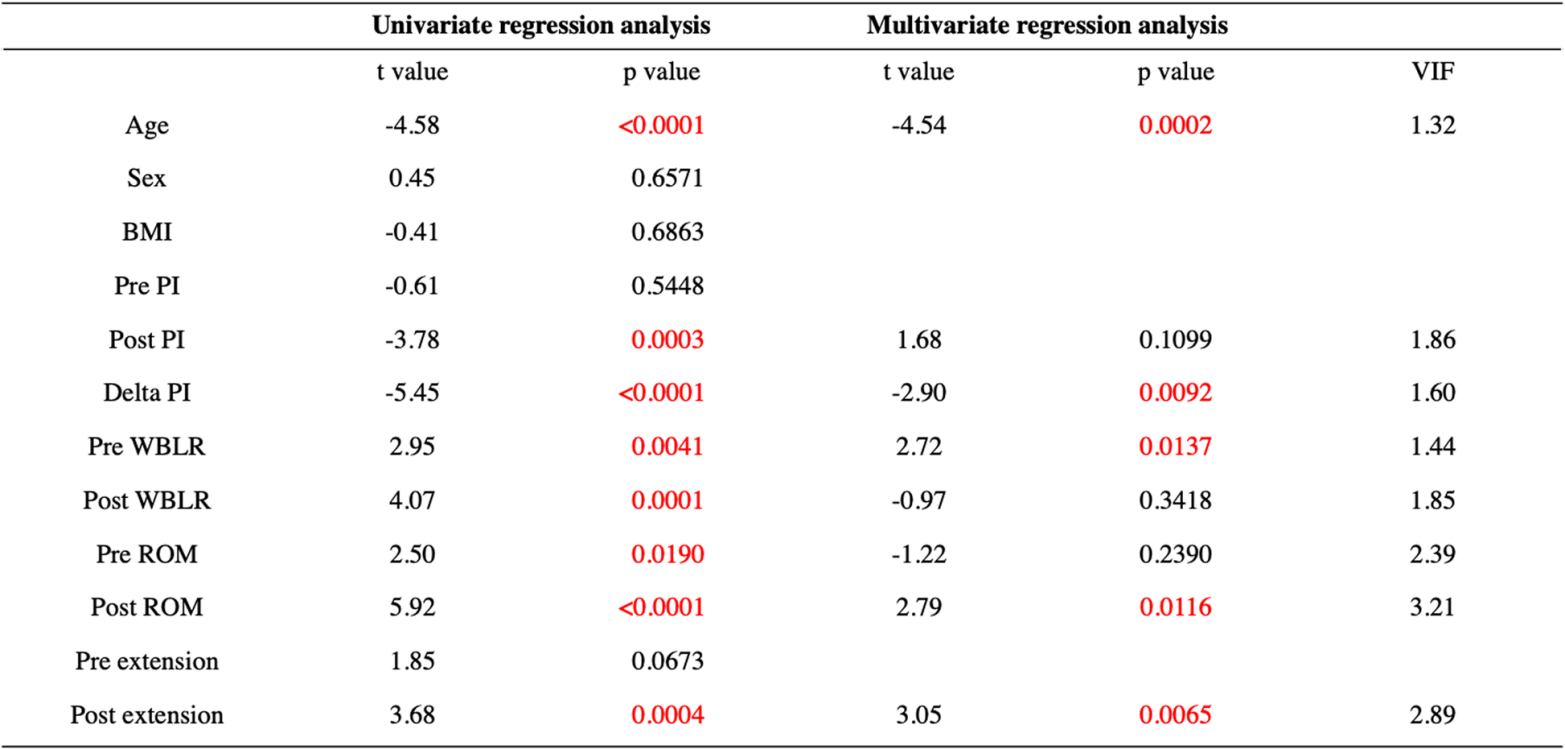
Regression analyses of the factors affecting the postoperative clinical outcome

*PI* pelvic inclination, *WBLR* weight bearing line ratio, *ROM* range of motion (flexion – extension)

**Table 4:**
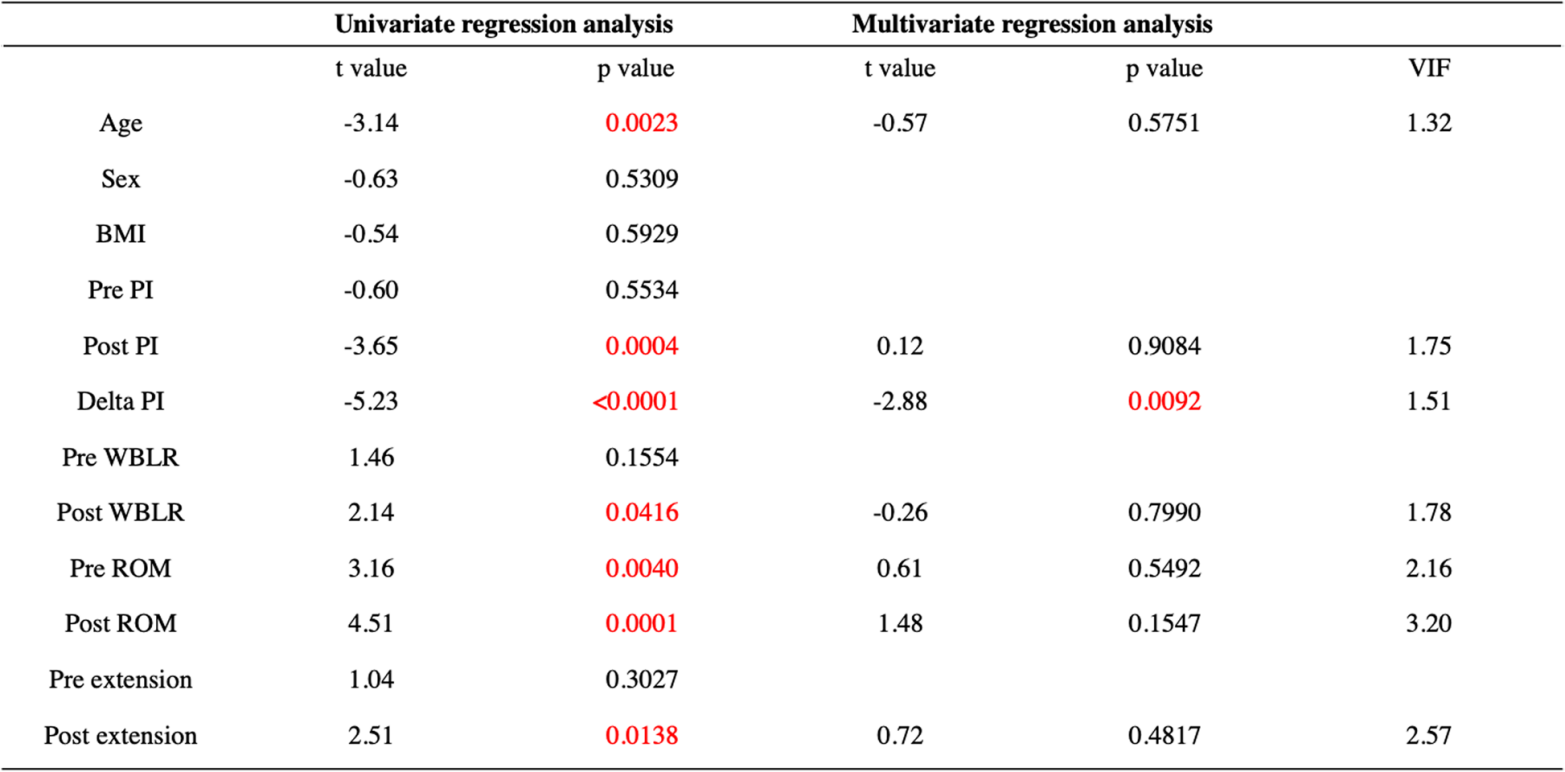
Regression analyses of the factors affecting the improvement of clinical outcome

*PI* pelvic inclination, *WBLR* weight bearing line ratio, *ROM* range of motion (flexion – extension)

**Table 5:**
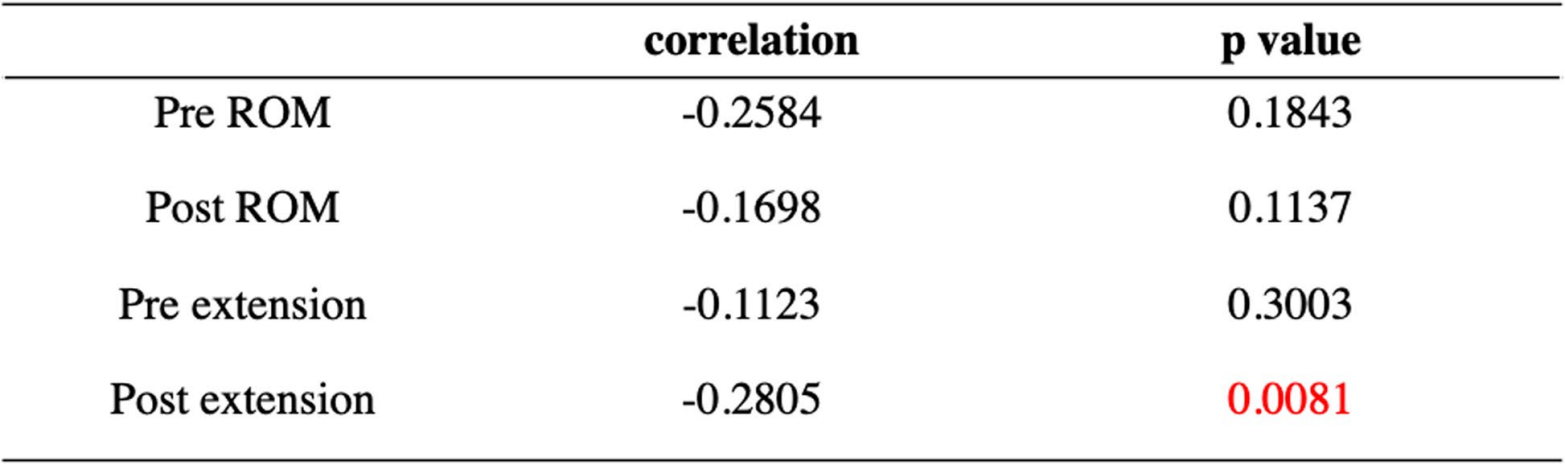
Correlation between changes of pelvic inclination and knee motion

*ROM* range of motion (flexion – extension)

## Discussion

The most important finding of this study was that age and the progression of pelvic retroversion were negatively correlated, whereas mild varus knee deformity and preserving postoperative ROM, especially extension, were indicated to have positive effects on the postoperative clinical outcome. Particularly, the progression of pelvic retroversion, which was correlated with postoperative knee flexion contracture, was a critical factor that deteriorated the improvement of clinical outcomes with OWHTO.

OWHTO generally provides good clinical outcomes, especially for active patients. Although Floerkemeir et al. did not identify a clear effect of aging on the clinical outcomes after OWHTO, the demographics of their study group was different from ours, with a mean age of 49.3 (range, 18 to 84) years and with a predominance of males (68.9%) [3]. In contrast, Pannell et al. identified aging as a risk factor for poor clinical outcome after OWHTO among patients with a mean age of 66.5 years and with a predominance of females [18]. Goshima et al. compared outcomes of OWTHO between older patients (mean age, 68.7 years) and younger patients (mean age, 56.2 years) and concluded that age does not affect clinical outcomes after OWHTO [4]. Through multivariate regression analysis, the current study showed that age was one of the predisposing factors that affect the postoperative outcome. One of the reasons for the difference with the previous study regarding the aging effect on OWHTO might be the postoperative WBLR. The WBLR in Goshima’s study was almost 66%, which was larger than the WBLR in our study at 57.9%. Recently, it has been recommended that the weight-bearing line should be close to neutral for patients who perform high-demand sports activities [8, 20]. The target weight-bearing line after OWHTO should be higher to achieve a good outcome, especially in elderly patients, based on previous results [4].

The Lysholm score was used to evaluate the postoperative clinical outcome of OWHTO because this scoring system includes the assessment of limping, potential support, locking, instability, pain and swelling of the knee, which is suitable for evaluating the activity of OWHTO patients. In addition, this also evaluates the ability to climb stairs and squat, which might be useful for evaluating general activity level, where over 84 points were considered a good clinical outcome [13]. Although the average score was 81.5 points in this study, which did not reach a good level for postoperative OWHTO, the average age of 72.4 years had affected this score because the preoperative baseline of the Lysholm score might be lower compared with young patients. That is why we had analyzed not only postoperative but also the improvement of Lysholm score with multivariate regression analysis in Tables 3 and 4.

A previous study had reported that individuals > 50 years of age with severe knee OA were strongly related to sagittal plane lumbopelvic malalignment and disability-related low back pain [26]. In the current study, progressive pelvic retroversion (increasing delta PI) was the critical factor for the deterioration of clinical outcomes after OWHTO. Furthermore, preserving postoperative ROM, especially extension, was also shown as an essential factor for OWHTO outcome. Tsuji et al. had reported that pelvic retroversion was associated with the development of a knee flexion contracture [25], and the current study showed a significant correlation between the progression of pelvic retroversion and loss of extension (flexion contracture). This is considered that progression of pelvic retroversion, especially for elderly patients, is associated with knee flexion in standing, where knee flexion is the last compensatory mechanism to enable standing balance [15]. These findings indicate that progressing pelvic retroversion should be carefully considered among elderly patients who undergo knee preservation surgery, as the progression of pelvic retroversion can lead to poor clinical outcomes within a short follow-up period after OWHTO. The effects of other factors on clinical outcomes should also be carefully considered in sagittal and axial view; Posterior tibial slope and patellofemoral OA might be associated with flexion contracture and poor clinical outcome after HTO [16]. Uni-compartment arthroplasty (UKA) and other types of HTO, such as hybrid HTO or opening-wedge distal tubercle osteotomy [6, 17], might be considered for patients with symptoms of medial compartment OA who have a pelvic retroversion.

In the current study, PI and lower limb alignment were evaluated using whole-leg radiographs in standing positions. Generally, pelvic inclination and global alignment should be evaluated using several sagittal plane parameters [22]. However, PI quantified using Schwartz’s method is easy to measure with high intra-observer and inter-observer reliability, indicating that this might be one of the options for evaluating the correlation between pelvic inclination and lower limb alignment with standing position with alternatively.

To the best of our knowledge, this is the first report to show that the progression of pelvic retroversion is a critical factor for poor clinical outcomes after OWHTO among elderly patients. Therefore, we believe that information on sagittal alignment might be useful to explain the clinical outcomes of osteotomy and patient satisfaction, especially for elderly patients. In order to predict the progress of pelvic retroversion after OWHTO, it is necessary to consider the related factors for change of global alignment, such as lumber compression fractures associated with osteoporosis and muscle weakness around the hip joint.

The limitations of our study need to be acknowledged. First, we performed a retrospective, non-randomized, sequential review of a series of patients. Second, evaluation of PI, using whole-leg standing radiography, might not be sensitive to the correction in measured PI after OWHTO. However, we note that Schwartz’s method that we used is simple and has high intra- and inter-observer reliability. Furthermore, this method used the intra-individual change in PI from pre-and post-OWHTO, which might be less affected by the sensitivity of whole-leg standing radiographs. Third, X-ray analysis is the only procedure to evaluate the essential factors. MRI and second-look arthroscopy might have the potential to detect other associated factors affecting clinical outcomes.

In conclusion, although OWHTO is an attractive joint preservation surgery, the age and the progression of pelvic retroversion were associated with loss of knee extension (flexion knee contracture) and critical factors for poor postoperative clinical outcome after OWHTO. Therefore, care should be taken for the progression of pelvic retroversion after OWHTO in patients aged over 65 years because it deteriorates the clinical outcome by inducing the knee flexion contracture as a compensatory mechanism for the balance of sagittal alignment.

## Abbreviations

BMI: Body mass index
FTA: Femorotibial angle
K/L: Kellgren-Lawrence
OA: Osteoarthritis
OWHTO: Opening-wedge high tibial osteotomy
PI: Pelvic inclination
WBLR: Weight-bearing line ratio
